# Editorial for the Special Issue “Advances in Clinical Diabetes, Obesity, and Metabolic Diseases”

**DOI:** 10.3390/medicina61040595

**Published:** 2025-03-26

**Authors:** Yuzuru Ohshiro, Kunimasa Yagi, Yasuhiro Maeno

**Affiliations:** 1 Department of Internal Medicine, Omoromachi Medical Center, 1-3-1 Uenoya, Naha 900-0011, Okinawa, Japan; 2 School of Medicine, Kanazawa Medical University, 1-1 Daigaku, Uchinada 920-0293, Ishikawa, Japan; yagikuni@icloud.com; 3 Comprehensive Internal Medicine, Shiga University of Medical Science, 255 Gochi-cho, Higashiomi 527-8505, Shiga, Japan; maeno@belle.shiga-med.ac.jp; 4 Diabetes & Endocrinology Department, National Hospital Organization Higashi-ohmi General Medical Center, 255 Gochi-cho, Higashiomi 527-8505, Shiga, Japan

Diabetes, obesity, and metabolic diseases are posing significant challenges to healthcare systems globally. These conditions contribute to increased morbidity and mortality, so continuous advancements in therapeutic strategies and technologies are crucial. Over the past year or so, our Special Issue “Advances in Clinical Diabetes, Obesity, and Metabolic Diseases” has served as a platform for the sharing of novel interventions, innovative technologies, and real-world data that contribute to improved patient care. This Editorial will provide an overview of key developments in this field, highlight the contributions of this Special Issue, and outline future research directions.

Remarkable progress has been made in the management of diabetes and metabolic disorders over the last decade. Novel pharmacological treatments, such as weekly insulin [1], dual glucagon-like peptide (GLP)-1 and gastric inhibitory polypeptide (GIP) receptor agonists [2], and triple-combination therapies (GLP-1, GIP, and glucagon receptor agonists) [3], have been used in clinical practice. The emergence of oral GLP-1 receptor agonists has increased convenience for patients by enhancing their compliance and glycemic control [4]. Despite these advancements, critical gaps remain in our understanding of long-term cardiovascular outcomes, patient adherence, and real-world effectiveness in relation to these novel therapies [5]. Additionally, further evaluations are required regarding the integration of technology, including artificial intelligence and digital health monitoring, into diabetes management to optimize patient outcomes [6].

This Special Issue includes many high-quality studies, several of which stand out for their significant contributions to the field. Yagi et al. [7] investigated the effect of a robot-assisted diabetes self-management monitoring system and demonstrated significant improvements in glycemic control. Their findings highlight the potential of robotic systems in supporting diabetes care professionals and enhancing patient engagement. The number of patients with diabetes continues to increase worldwide, with notable increases in Asia, the Middle East, and Africa. Economic growth in these regions has led to changes in dietary habits and reduced physical activity, contributing to a sharp increase in the prevalence of diabetes. Simultaneously, access to diabetes specialists and appropriate treatment remain insufficient in these areas, leaving many patients without adequate care [8]. The study by Yagi et al. provides valuable insights into addressing this critical issue.

Tariq et al. [9] explored the association between telomere length and aging determinants in patients with type 2 diabetes. Their study challenged the notion that telomere length is the sole marker of biological aging by revealing significant correlations between telomere length and factors such as hypertension, smoking, and stress. These insights underscore the need for multidimensional assessments of age-related diabetes.

In another important study, Graňák et al. [10] examined the role of physical activity in preventing post-transplant diabetes mellitus. Their findings suggest that regular exercise significantly improves glucose tolerance outcomes after kidney transplantation, supporting the incorporation of structured exercise programs into post-transplant care protocols.

In another key study, Vuković et al. [11] investigated the neurometabolic alterations in obesity using cerebral multivoxel magnetic resonance spectroscopy. Their study revealed significant negative correlations between obesity markers and brain metabolites involved in cognitive and emotional processing, with hyperinsulinemia emerging as a critical factor affecting neurometabolic health. These findings emphasize the need for targeted metabolic interventions.

As the research on diabetes, obesity, and metabolic diseases continues to evolve, several key areas will require further investigation. The development of personalized medical approaches will enable more tailored treatment strategies by enabling the consideration of genetic, metabolic, and behavioral factors that influence individual responses to therapy. Additionally, expanding the roles of artificial intelligence, wearable devices, and telemedicine can enhance real-time monitoring and personalized interventions. Further studies are needed to evaluate the long-term cardiovascular outcomes of GLP-1 and GIP receptor agonists beyond glycemic control. Moreover, a deeper understanding of the complex interactions among obesity, insulin resistance, and neurodegeneration is essential in developing targeted therapeutic interventions for metabolic brain dysfunction.

This Special Issue provides valuable insights into the evolving landscapes of diabetes, obesity, and metabolic diseases. The studies featured here contribute to bridging knowledge gaps and advancing clinical applications. However, continued efforts are essential to address remaining challenges and enhance patient outcomes. We extend our gratitude to all the contributing authors, reviewers, and researchers who have enriched this collection with their expertise and dedication. As this field continues to develop, we anticipate that further ground-breaking discoveries will shape the future of metabolic disease management.

## Abbreviations

The following abbreviations are used in this manuscript:

GLP	glucagon-like peptide
GIP	gastric inhibitory polypeptide

## References

[1] Rosenstock J., Bain S.C., Gowda A., Jódar E., Liang B., Lingvay I., Nishida T., Trevisan R., Mosenzon O. (2023). Weekly Icodec versus Daily Glargine U100 in Type 2 Diabetes without Previous Insulin. N. Engl. J. Med..

[2] Salmen T., Potcovaru C.G., Bica I.C., Giglio R.V., Patti A.M., Stoica R.A., Ciaccio M., El-Tanani M., Janež A., Rizzo M. (2024). Evaluating the Impact of Novel Incretin Therapies on Cardiovascular Outcomes in Type 2 Diabetes: An Early Systematic Review. Pharmaceuticals.

[3] Kwon J., Thiara D., Watanabe J.H. (2025). Oral versus subcutaneous semaglutide weight loss outcomes after two years among patients with type 2 diabetes in a real-world database. Expert Rev. Endocrinol. Metab..

[4] Jiang Y., Zhu H., Gong F. (2025). Why does GLP-1 agonist combined with GIP and/or GCG agonist have greater weight loss effect than GLP-1 agonist alone in obese adults without type 2 diabetes?. Diabetes Obes. Metab..

[5] An X., Sun W., Wen Z., Duan L., Zhang Y., Kang X., Ji H., Sun Y., Jiang L., Zhao X. (2025). Comparison of the efficacy and safety of GLP-1 receptor agonists on cardiovascular events and risk factors: A review and network meta-analysis. Diabetes Obes. Metab..

[6] Anwar A., Rana S., Pathak P. (2025). Artificial intelligence in the management of metabolic disorders: A comprehensive review. J. Endocrinol. Investig..

[7] Yagi K., Inagaki M., Asada Y., Komatsu M., Ogawa F., Horiguchi T., Yamaaki N., Shikida M., Origasa H., Nishio S. (2024). Improved glycemic control through robot-assisted remote interview for outpatients with type 2 diabetes: A pilot study. Medicina.

[8] Sun H., Saeedi P., Karuranga S., Pinkepank M., Ogurtsova K., Duncan B.B., Stein C., Basit A., Chan J.C.N., Mbanya J.C. (2022). IDF diabetes atlas: Global, regional and country-level diabetes prevalence estimates for 2021 and projections for 2045. Diabetes Res. Clin. Pract..

[9] Tariq J.A., Mandokhail K., Sajjad N., Hussain A., Javaid H., Rasool A., Sadaf H., Javaid S., Durrani A.R. (2024). Effects of age and biological age-determining factors on telomere length in type 2 diabetes mellitus patients. Medicina.

[10] Graňák K., Vnučák M., Beliančinová M., Kleinová P., Blichová T., Pytliaková M., Dedinská I. (2024). Regular physical activity in the prevention of post-transplant diabetes mellitus in patients after kidney transplantation. Medicina.

[11] Vuković M., Nosek I., Slotboom J., Medić Stojanoska M., Kozić D. (2024). Neurometabolic profile in obese patients: A cerebral multi-voxel magnetic resonance spectroscopy study. Medicina.

